# Review of "Advanced Methods and Tools for ECG Data Analysis", by Gari D. Clifford, Francisco Azuaje and Patrick E. McSharry (Editors)

**DOI:** 10.1186/1475-925X-6-18

**Published:** 2007-05-25

**Authors:** Ivan Dotsinsky

**Affiliations:** 1 Center of Biomedical Engineering, Bulgarian Academy of Sciences, 105, Acad. G. Bonchev Str., 1113 Sofia, Bulgaria

The automated analysis of the electrocardiogram (ECG) is an important part of the general problem of interpretation of biomedical signals. First, ECG signal evaluation is known to be one of the most informative and significant tools not only for cardiac diagnostics but also for correlative examination of the state of other systems in the body. Secondly, many approaches developed for analysis of the ECG may be applied to different biomedical signals.

Although the title of this book emphasizes the analysis, the content also includes important topics related to signal preprocessing. Really, these categories are used synonymously to some extent, perhaps because of the fuzzy border between signal preprocessing and analysis. Strictly speaking, ECG analysis aims to find out the best correlation between a set of measured parameters and one of statistically created diagnostic patterns, while preprocessing deals with the improvement in signal-to-noise ratio. Some functions, such as QRS detection, may be considered to be either preprocessing or analysis, but the more important issue is that correct ECG analysis can not be accomplished with corrupted input signals.

This book will be of great interest to clinicians, engineers and scientistswho wish to apply modern processing techniques to the analysis of ECG signals. It is clearly not possible to cover all of the problems in this vast field in detail. However, the book offers an almost complete and competent presentation of signal acquisition, linear and nonlinear filtering, wave detection, parameter extraction, signal classification related to the ECG, together with some specific approaches to detection of rhythm abnormalities. I praise the authors highly for their competency, skill and energy in creating a presentation of these topics at a high level.

The book is well organized. The first of its 13 chapters acquaints the reader with the necessary physiological knowledge on the excitable cell membrane and propagation of action potentials leading to rhythmic myocardial contractions. The authors describe complex neuromuscular interactions logically and very clearly, providing medical background to allow the reader to understand the genesis and the specificity of the electric signals in the heart accompanying its function as a pump. Attention is paid to the spontaneous cycle of depolarization and repolarization, which is initiated by different pacemakers: the sino-atrial node activated in normal cases by the sympathetic and parasympathetic portions of the autonomic nervous system, or other substituting parts of the myocardium leading in 'emergency' cases and/or propagation failures to premature atrial and ventricular beats. The authors describe rhythm abnormalities and disorders such as bradycardia, tachycardia, arrhythmia, atrial flutter and fibrillation, atrioventricular blocks, as well as the quickly-fatal condition of circulatory arrest due to ventricular fibrillation. Finally this chapter discusses the projections of the cardiac vector on planes (vectorcardiographic loops) and lines (usually the standard 12 leads), and relates the waves, intervals and segments of the acquired ECG from electrodes on the body surface to corresponding phases of the cardiac contraction cycle. One paragraph (1.3.5) points out the usefulness of the systematic approach to clinical ECG analysis

The second chapter deals with techniques of ECG acquisition. The widely used instrumentation amplifier is discussed in the context of suppressing the common mode voltage due to the power-line interference and amplifying the useful differential signal. The authors recommend against the use of 50 and 60 Hz notch filters since they affect signal components. A small misfit is observed in Figs. 2.2 and 2.3 where the output of the active grounding circuit GND from Fig. 2.3 is named input C in Fig. 2.2. The circuit diagram includes an optically isolated differential amplifier, which gives additional patient protection against unwanted dangerous power-line leakage currents.

The quasi DC component is eliminated by an eighth-order Bessel high-pass filter with a cutoff at 0.1 Hz, having optimal phase response with negligible phase distortion. Usually this is done by first-order high-pass filter [1] implemented by simple RC circuit at 0.05 Hz (τ = 3.2 s) to reduce the distortions around 0.3 Hz, accepted as the low frequency limit of the informative signal range [2]. In my opinion, a presentation of other approaches to drift suppression would be beneficial to the reader. Some of them have been proposed many years ago and continuously improved later. An example is bi-directional digital high-pass filtering with the second filtering pass applied in time-reversed direction [3]; another example is high-pass filtering by linear-phase low-pass filters to average out the drift, which is then subtracted from the ECG signal [4].

Special attention is paid to low-pass filtering of the signal, and the use of oversampling to avoid aliasing errors.

Chapter 2 also discusses appropriate storage formats for the ECG, includingthat applied by the MIT-BIH database, which is widely used to evaluate the efficiency of QRS detection algorithms. The authors present the formulae for calculating the sensitivity and positive predictivity of a QRS detection algorithm, based on number of true positive, false positive and false negative beats in an ECG recording.

Chapter 3 is entitled 'ECG statistics, noise, artifacts, and missing data' and discusses numerous topics. The authors present standard clinical features of the ECG and describe the parameters of the ECG: parameters of the QRS complex, RR interval length, PR and QT intervals. In addition, the authors provide very interesting and useful information about the interpretation of the so-called QT hysteresis. They also describe some aspects of the Heart Rate Variability in time and frequency domains, which is an important part of the analysis of the cardiac rhythm and the consequent diagnosis of the heart.

Also in Ch. 3 the authors give interesting information about the rarely applied spectral and cross-spectral ECG analysis. The authors show that the cross spectral coherence between pair of leads with sinus rhythm is higher than 0.9 at the frequency range between 1 and 10 Hz. In the case of ventricular arrhythmia, the QRS complex becomes broader but spectral separation between sinus rhythm and ventricular tachycardia is difficult to detect, except in the case of ventricular flutter. The detection of atrial arrhythmia is hampered by the relatively small deviations in R-R time intervals. In this paragraph the accepted frequency range of the diagnostic ECG is stated to be from 0.05 to about 100 Hz, but for detection of ventricular late potentials the authors recommend a higher frequency limit of 500 Hz, while for sleep apnea diagnostics the lowest frequency is shifted down to 0.02 Hz.

Chapter 4 describes models for ECG and RR interval processes. Among them I would like to emphasize the synthetic ECG generation and the Integral Pulse Frequency Modulation Model, developed originally for investigation of discrete series of events, which is largely used in the theory of the Heart Rate Variability interpretation.

Chapters 5 and 6 are devoted to linear and nonlinear filtering methods, both of great importance for accurate analysis of the ECG. Numerous techniquesare presented: Wiener and Wavelet Filtering, Principal Component Analysis, Neural Networks, Lyapunov Exponents, Entropy, Model-based Filtering and so on, all of which are widely accepted approaches for noise suppression. However, especially for elimination of power-line interference, these all have the drawback of affecting the frequency components in the signal. This phenomenon might be observed explicitly in all Figures if they contained successive graphics of: input 'clean' signal, this signal superimposed by noise, processed signal, difference between 'clean' and processed signal. Such approach is implemented to some extent in Fig. 6.6, where it can be seen that the amplitudes of the residual noise are not acceptable. It is a pity that the authors in Chapter 6 have not included brief information about the subtraction procedure [5], which preserves the frequency content of the signal while providing almost total cancellation of power-line interference regardless of its amplitude and frequency modulation.

Chapter 7 provides a very interesting presentation of the T-wave alternans phenomenon. Special attention is paid to measurement techniques, which are critical to the correct assessment of this phenomenon.

Chapter 8 competently describes almost all known techniques for estimating the respiratory frequency in ECG signals. A first group of algorithms for this purpose is based on beat morphology: amplitude of the QRS complex in single lead recordings, QRS area in multilead recordings, and electrical axis rotation in vectorcardiographic loops. Other methods described in this chapter are based on heart rate variation; adaptive filtering is also presented. In the last case the authors unfortunately exchange the primary and reference inputs in Fig. 8.13. A good presentation of the background of this adaptive filtering technique may be found in [6].

Chapter 9 is devoted to the very important topic of feature extraction. A short paragraph describes preprocessing necessary for this task, in which the authors recommend use of a Butterworth 4- or 6-pole low-pass filter with a cut-off of 45 to 55 Hz for rejection of high-frequency noise. However, asa result the QRS parameters shown in Fig. 9.2 can not be measured adequately. By contrast, the authors correctly present the problem of baseline wander. Many readers would find the appendix on the Karhunen-Loève Transform (Principal Component Analysis) to be very useful.

Chapter 10 discusses the important topic of ST analysis. This requires close attention by any clinician and engineer involved in developing methods for automatic interpretation of the ECG. The authors present an interesting comparison of the performance of some ST analyzing methods.

The Probabilistic Modeling Approach to interpretation of the ECG is discussed in detail in Chapter 11. The authors underline the ability of this approach to incorporate prior knowledge, e.g. to learn ECG data sets annotated by experts. Further, the authors discuss use of hidden Markov models in supervised and unsupervised learning by computer algorithms and the use of the wavelet transform for ECG encoding.

The last two chapters deal with supervised and unsupervised methods of classification of ECG. Examples of supervised methods are neural networks and support vector machines. The preprocessing methods used for feature generation are the higher-order statistics and the Hermite basis functions, which offer better presentation of QRS complexes that are characterized by large differences in duration. Chapter 13 introduces clustering-based techniques and use of self-adaptive neural networks, and offers a discussion of the limitations and future opportunities for unsupervised classification of the ECG.

My conclusion after reading the book is that the chapters are individually well organized but there is some overlapping between chapters. In my opinion, this is not necessarily a drawback since the multiple presentations of methods and approaches for ECG analysis occur within different contexts. Many readers will find the repeated acquaintances of background knowledge to be useful, especially when they are provided by different authors in the different chapters. The quality of the Figures, Graphics and Tables is excellent. The lists of references at the ends of the chapters are well selected and representative.

Finally, I would like to share that I found this book interesting and hard to put down, and read it almost at one sitting
